# Supplementary material for: Actin Isovariant ACT7 Modulates Root Thermomorphogenesis by Altering Intracellular Auxin Homeostasis
Source: Int J Mol Sci. 2021 Jul 20;22(14):7749. doi: 10.3390/ijms22147749 (PMC8306570; doi:10.3390/ijms22147749)
Supplement: Supplementary file 1 [file ijms-22-07749-s001.zip › ijms-1277504-supplementary.pdf]

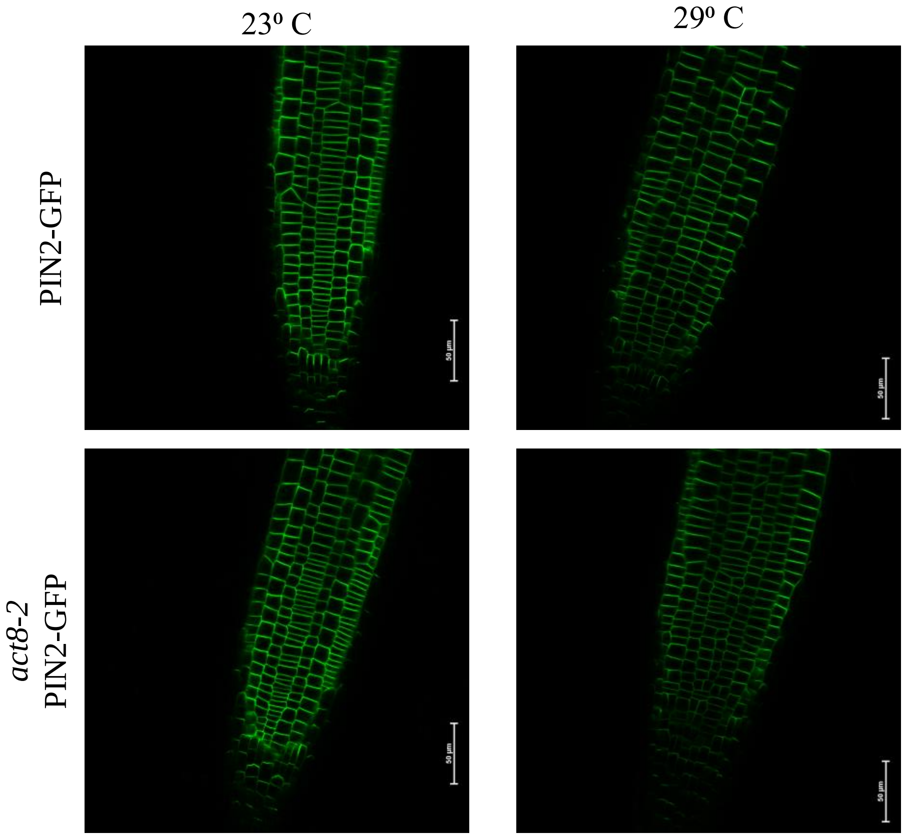

Figure S1 : Effect of prolonged moderate high temperature on PIN2 abundance in *act8-2* roots. Five-day-old light grown seedlings were transferred to new agar plates and subjected to high-temperature treatment (29º C) for 72 hours before imaged with confocal microscopy using the same confocal settings. Images are representative of 15 to 20 seedlings obtained from three independent experiments.

Bars = 50 µm.

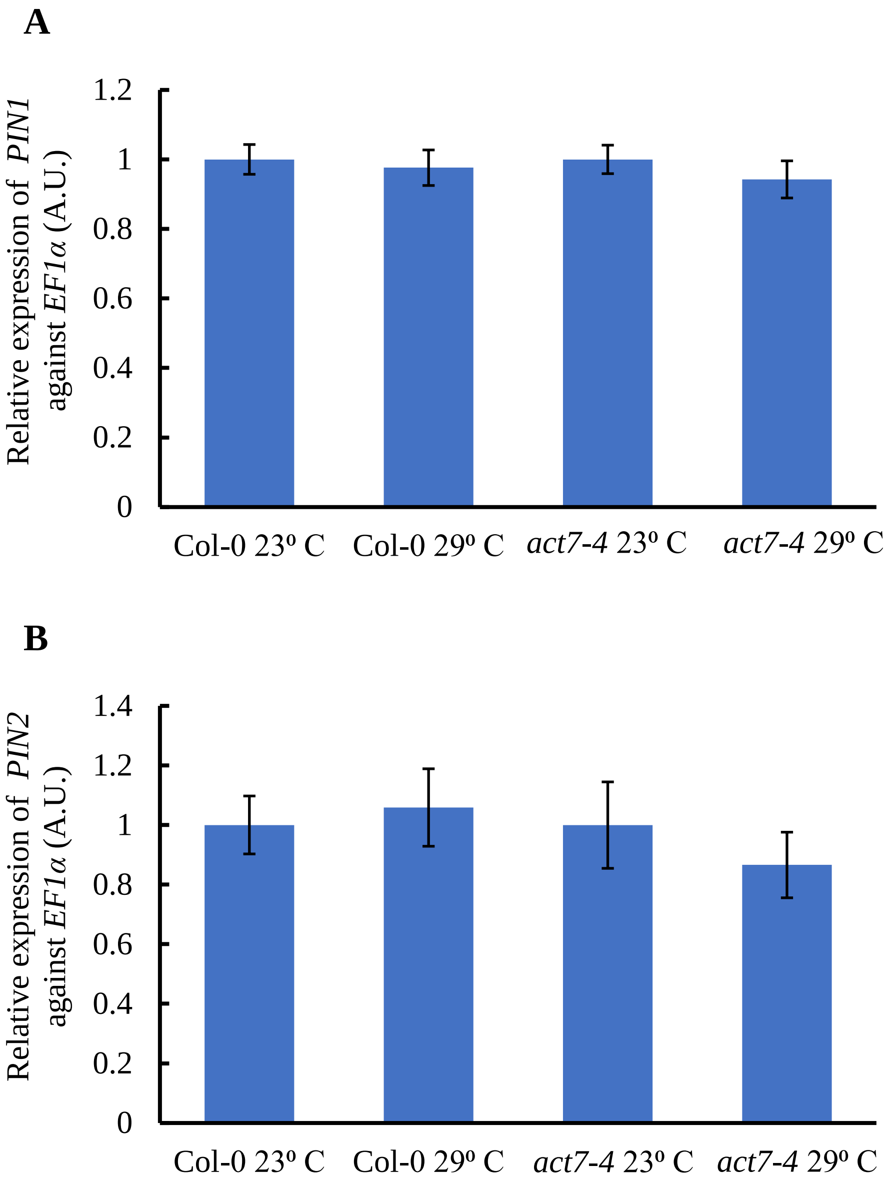

Figure S2. Expression of *PIN1* and *PIN2* are not changed at transcriptional level under prolonged moderate high temperature in Col-0 and *act7-4*.

(A) *PIN1* expression under prolonged high temperature in Col-0 and *act7-4*. (B) *PIN2* expression under prolonged high temperature in Col-0 and *act7-4*.

Five-day-old light grown seedlings were transferred to new agar plates and subjected to high-temperature treatment (29º C) and control (23º C) for 72 h before collecting RNA for semi-quantitative RT-PCR analysis. All the data were normalized against *EF1α*. The data were obtained from three biological replicates. Vertical bars in the graph represent mean ± SE.

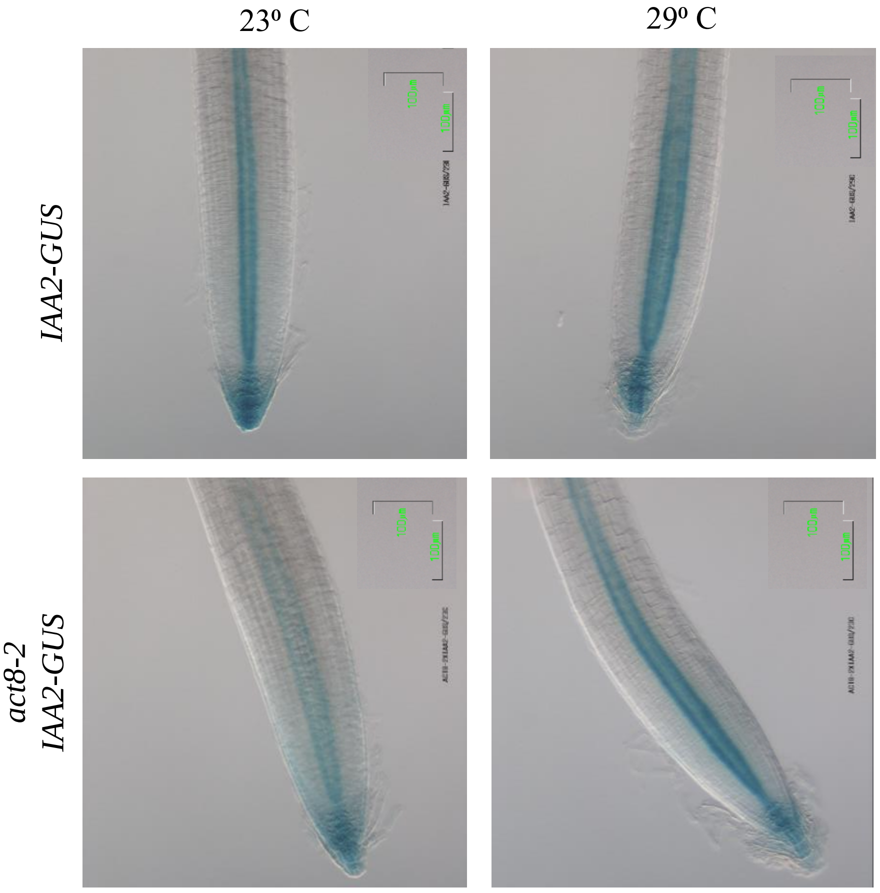

Figure S3. Auxin distribution in *act8-2* roots under prolonged moderate high temperature. Five-day-old *IAA2-GUS* and *act8-2 IAA2-GUS* seedlings were transferred at 23º C and 29º C for 72 hours. Seedlings were stained in a buffer containing 1 mM X-gluc for 1 h at 37º C and cleared for photography. These are representative images stained in at least three separate experiments.
Bars = 100µm.

Table S1. Primer list used to perform PCR.

PRIMER	SEQUENCE
PIN1 F	ATCTTCACATGTTTGTGTGG
PIN1 R	TCGTCTTTGTTACCGAAACT
PIN2 F	GGAGACGGCTGGTTCAATTA
PIN2 R	ACCACCACGTGTAGCTTTCC
EF1α F	CTTGCTTTCACCCTTGGTGT
EF1α R	TCCCTCGAATCCAGAGATTG
